# A Rare Case of Adrenocortical Carcinoma Manifesting as a Pulmonary Embolism

**DOI:** 10.7759/cureus.52929

**Published:** 2024-01-25

**Authors:** Madeline Vithya Barnaba Durairaj, Kasey Shallenburg, Neeraj Ashri, Punam Rajput

**Affiliations:** 1 Internal Medicine, UNC Health Blue Ridge, Morganton, USA; 2 Diabetes and Endocrinology, Atrium Health, Kings Mountain, USA

**Keywords:** cushing's syndrome, hirsutism, virilization, pulmonary embolism (pe), adrenocortical carcinoma (acc)

## Abstract

Adrenocortical carcinoma (ACC) is a very rare malignancy with a poor prognosis. It is predominantly noted in the fourth to fifth decades of life and is more common in White females. ACC is most commonly detected as an incidental finding but may have other presentations, such as rapid-onset Cushing's syndrome or pulmonary embolism. In the current case, ACC was incidentally observed in a 24-year-old female during imaging, and the patient later developed a pulmonary embolism. Lab investigations were suggestive of hypercortisolism along with hyperandrogenism. Following preoperative treatment with beta-blockers, metyrapone, and therapeutic anticoagulation, she underwent left radical nephrectomy with left open adrenalectomy and inferior vena cava (IVC) resection and reconstruction. Surgery was uncomplicated, and she was discharged with plans for outpatient adjuvant chemotherapy. This case highlights the fact that a seemingly unprovoked pulmonary embolism may point to the possibility of an underlying occult malignancy and undetected ACC should be included in the differential diagnosis of such cases.

## Introduction

Adrenal incidentaloma is a common endocrine diagnosis that affects about 2% of the general population. It is defined as a mass lesion greater than 1 cm, detected on imaging studies that were done for an unrelated reason [1]. It is often seen unilaterally, but about 10-15% of cases have bilateral adrenal masses. Although rare, around 2-5% of the cases are subsequently confirmed to be primary adrenal carcinoma [2]. Adrenal masses greater than 4 cm are more likely to be adrenal carcinoma [1]. Other features pointing toward malignancy include an irregular border, inhomogeneity, <40% washout of contrast after 15 minutes, calcifications, and Hounsfield units >10 [3]. Apart from being detected incidentally, they can also present with rapid-onset Cushing’s syndrome and features of androgen excess [4]. Appropriate management of these incidentally detected tumors requires a thoughtful evaluation of their hormonal function.

Here, we present a rare case of adrenocortical carcinoma (ACC) with an unusual presentation of incidentaloma along with a coexisting pulmonary embolism. This case emphasizes the fact that ACC can have multiple presentations, including incidentaloma, rapid-onset Cushing's syndrome, virilization, and pulmonary embolism.

## Case presentation

**Figure 1:**
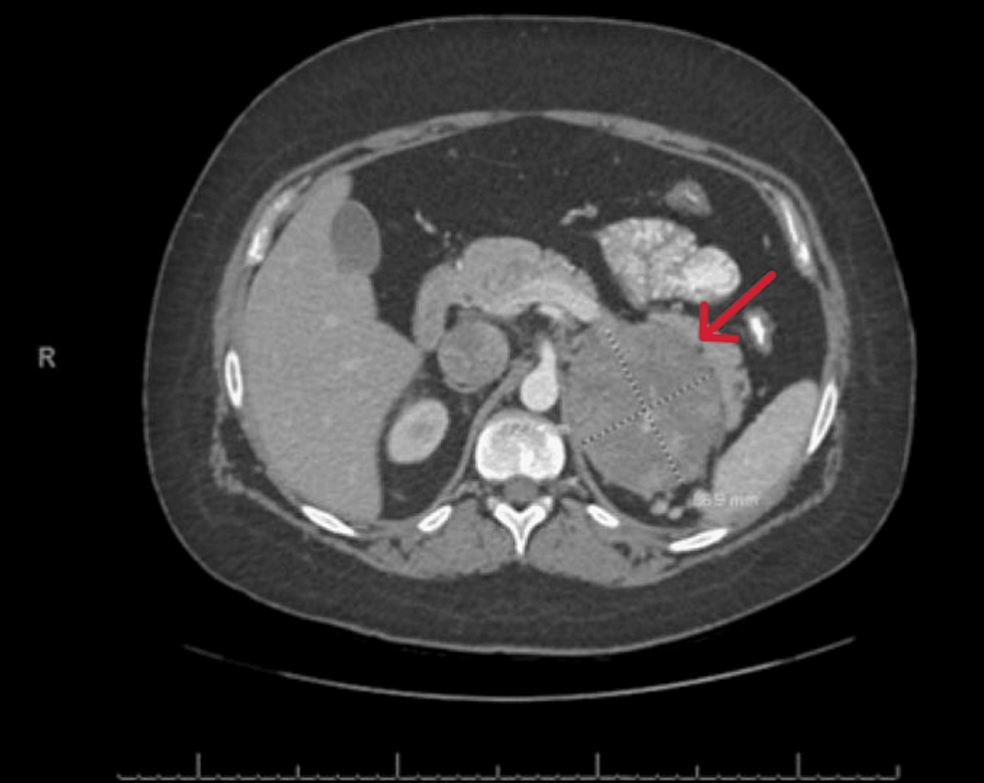
CT scan of the abdomen (axial view) with contrast showing the left adrenocortical carcinoma (red arrow).

**Figure 2:**
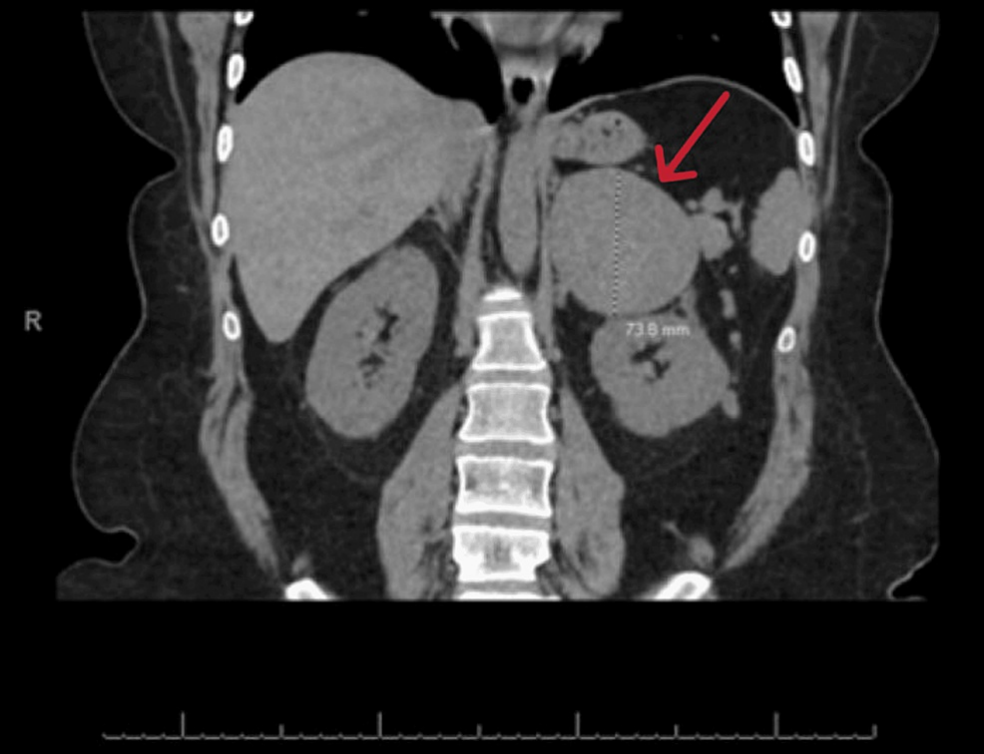
CT scan of the abdomen (coronal view) with contrast showing the left adrenocortical carcinoma (red arrow).

**Figure 3:**
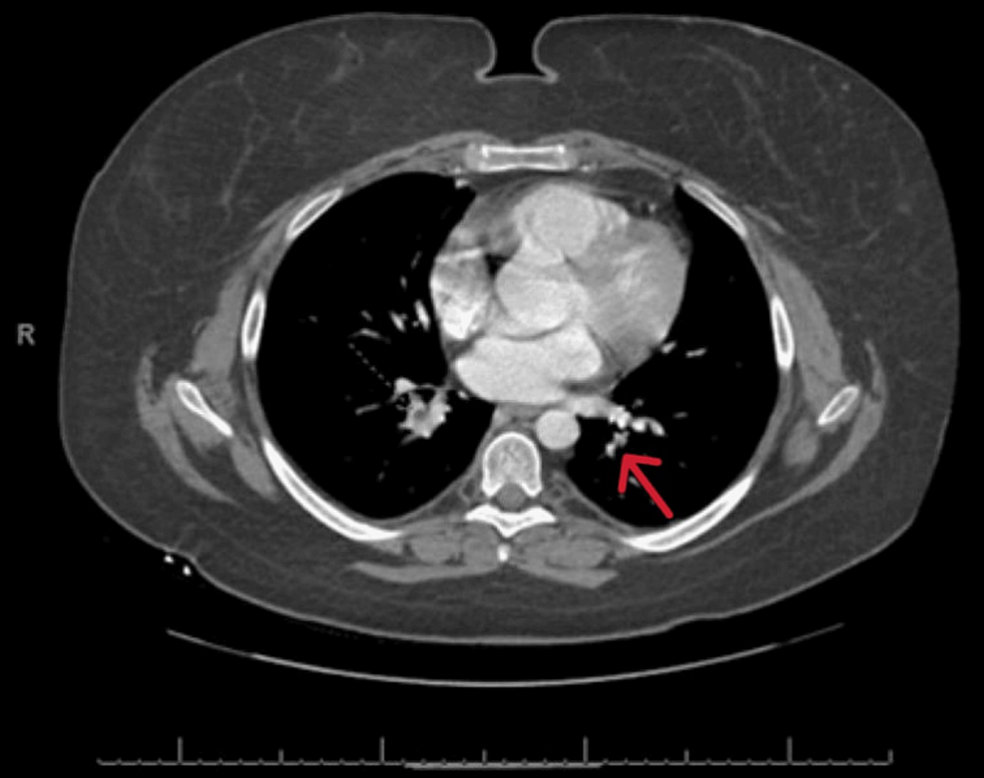
CT scan of the chest (axial view) with contrast showing the left pulmonary embolism (red arrow).

A 24-year-old African American female presented to the emergency department with acute onset of the left flank plain of one-day duration. Physical examination was unremarkable. Computed tomography (CT) scan of the abdomen with contrast showed two small calculi at the left uretero vesicular junction with mild left-sided hydroureter. Incidentally noted was a large rounded heterogeneously enhancing mass in the left suprarenal region measuring 8.7 x 7.4 x 7.3 cm, separate from the left kidney and abutting the spleen, a pancreatic tail with a direct extension into the left renal vein, and an inferior vena cava (IVC) with an uncertain cephalad extent (Figures 1, 2, 3).

She was diagnosed with left adrenal incidentaloma. While she was awaiting work-up for the same, she developed acne and an erythematous rash of the face along with hirsutism. She also reported a 60-pound weight gain over the last six months, secondary amenorrhea, and depression with anxiety. There was no history of exogenous steroid intake, palpitations, or intense headaches. Family history was negative for pituitary, parathyroid, and adrenal problems. On examination, her height was 167.6 cm and her weight was 131 kg, with a BMI of 46.65 kg/m^2^. Blood pressure was elevated (155/88 mm/Hg). Skin examination showed acanthosis nigricans along with erythematous rash and acne affecting the face. Supraclavicular fullness and dorsal cervical hump were also noted. Abdominal examination showed central obesity with striae. There was no evidence of clubbing, easy bruising, or hyperpigmentation of the oral mucosa or skin. Other systems were unremarkable.

Investigations revealed normal complete blood count and serum electrolytes. The urine pregnancy test was negative. The 24-hour urine-free cortisol and serum cortisol were high. Salivary cortisol was high on days 1, 2, and 3. Plasma adrenocorticotropic hormone (ACTH) was low. Serum aldosterone was low, plasma renin activity was normal, and the renin aldosterone ratio was normal. Dehydroepiandrosterone (DHEA) and testosterone levels were high (Table 1).

**Table 1:** Lab investigation results

Lab parameter	Patient's findings	Reference range
24-hour urine-free cortisol	539 mcg/24 hours	10-100 mcg/24 hours
Serum cortisol	33.1 mcg/dl	5-25 mcg/dl
Salivary cortisol - day 1	937 ng/dl	30-110 ng/dl
Salivary cortisol - day 2	980 ng/dl	30-110 ng/dl
Salivary cortisol - day 3	870 ng/dl	30-110 ng/dl
Plasma adrenocorticotropic hormone (ACTH)	<1.5 pg/ml	10-60 pg/ml
Serum aldosterone	<1 ng/dl	2-9 ng/dl
Plasma renin activity	2.85 ng/ml/hour	1.9-3.7 ng/ml/hour
Renin aldosterone ratio	<0.4	0-30
Dehydroepiandrosterone (DHEA)	838 pg/ml	101-688 pg/ml
Testosterone levels	112 ng/dl	15-70 ng/dl

**Figure 4:**
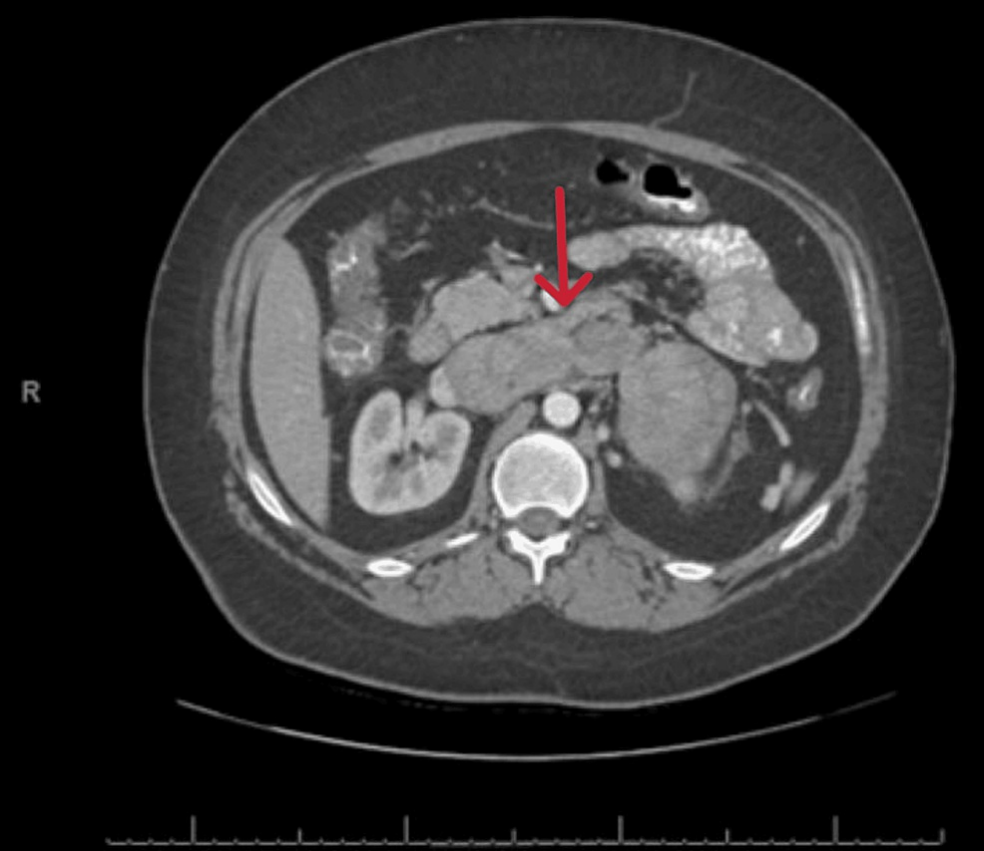
CT scan of the abdomen (axial view) with contrast showing the left adrenocortical carcinoma (red arrow) with extension into the inferior vena cava.

**Figure 5:**
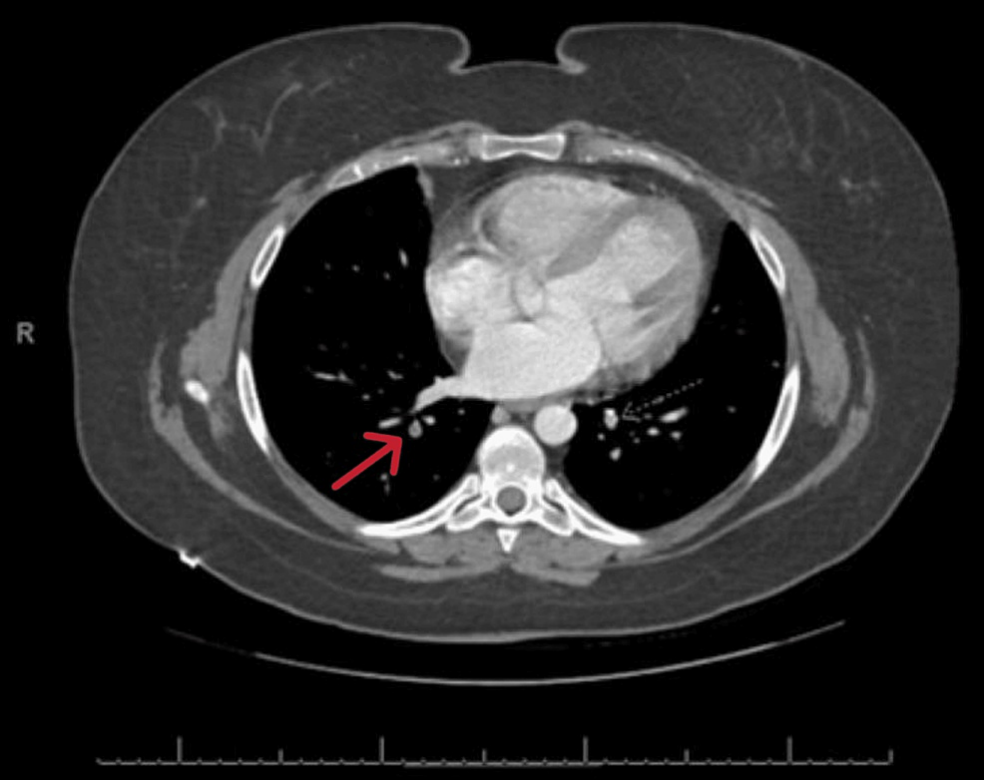
CT scan of the chest (axial view) with contrast showing the right pulmonary embolism (red arrow).

The 17-hydroxy progesterone, estrogen, plasma/urine metanephrine, and normetanephrine levels were noted to be normal. MRI of the adrenals revealed a large mass that completely replaced the left adrenal and measured up to 8.4 x 7.3 x 7.7 cm. There was a direct tumor invasion and expansion of the left renal vein and IVC with the craniocaudal extent of the tumor within the IVC measuring roughly 5.2 cm. There was also a small volume tumor extension into the superior aspect of the left gonadal/ovarian vein. Small cystic and necrotic components were noted within the primary mass and the renal vein component. A few weeks later, she presented with an acute onset of shortness of breath. She was found to be tachycardic and hypotensive. The chest X-ray was normal. Echo showed an ejection fraction of 55%, with no segmental wall motion abnormalities, and a mild right ventricular dilation with no valvular stenosis or regurgitation. CT chest with contrast showed a band of plate-like atelectasis seen within the left lower lobe of the lung along with pulmonary arterial filling defects within both lower lobes of the lung consistent with peripheral pulmonary embolism (Figures 4, 5). 

She underwent surgical resection of the left adrenal mass because of suspected ACC. Preoperatively, she was started on carvedilol, metyrapone, and therapeutic anticoagulation with enoxaparin. She underwent a left radical nephrectomy with left open adrenalectomy and IVC resection and reconstruction. Intraoperatively, a large left adrenal tumor was noted with adherence to the diaphragm posteriorly. A mobile tumor thrombus was noted within the left renal vein and IVC. Surgical pathology reported that the mass measured 9.5 cm in the greatest dimension, and malignant cells showed positive staining for calretinin, inhibin, CD56, mart-1, and synaptophysin. There was negative staining for CAM 5.2 (cytokeratin) and PAX-8. The maximum mitotic rate was 46 per 50 hpf. According to the revised Weiss criteria, the score was 4, and this mass exhibited a malignant behavior. There was no nodal spread noted (T4N0M0). The pre-op PET scan revealed no distant metastasis.

The patient was doing well symptomatically in the post-surgical follow-up after two weeks. She was treated with adjuvant chemotherapy mitotane and hydrocortisone in view of transient adrenal insufficiency.

## Discussion

Adrenal tumors are often detected incidentally. Mostly, they are adrenal adenomas, but ACC is quite rare with an incidence of approximately one to two per million population per year. Studies have shown a bimodal age of distribution with onset before the age of five and in the fourth to fifth decades of life [5]. It is more commonly noted in women and can be sporadic or associated with hereditary cancer syndromes [6]. Sixty percent of ACCs present with hormonal excess, mostly with Cushing’s syndrome, or a few mixed with virilization, due to excess of glucocorticoids and androgens. A clue to the diagnosis would be the rapid onset of Cushing’s syndrome [7]. A presentation of virilization as in the present case points to ACC rather than adenoma. The current case was unusual with multiple complications following detection of an incidentaloma.

The European Network for the Study of Adrenal Tumors recommends the following evaluations of even asymptomatic patients with adrenal tumors: fasting blood glucose, serum potassium, cortisol, corticotropin (ACTH), 24-hour urinary free cortisol, fasting serum cortisol at 8 AM following a 1 mg dose of dexamethasone at bedtime, adrenal androgens (DHEAS, androstenedione, testosterone, and 17-hydroxyprogesterone), and serum estradiol in men and postmenopausal women [8]. Adrenal carcinomas are typically associated with excessive amounts of adrenal steroid precursors and precursors of androgens and glucocorticoids [8]. This was seen in the current case with her elevated 24-hour urine-free cortisol, serum cortisol, salivary cortisol, DHEA, and testosterone levels. Other typical findings of ACCs that were also seen in our patient include low serum aldosterone, normal plasma renin activity, and low renin aldosterone ratio. Our patient’s laboratory investigations were consistent with those typically seen in ACCs.

Treatment depends on the staging of ACC, which makes it resectable or unresectable. Complete surgical resection remains the only curative option for ACC [9]. Stage I-III diseases are deemed to be surgically resectable. Preoperative complete hormonal assessment is important to manage the anticipated postoperative adrenal insufficiency. Our patient had T4N0M0 (stage III) disease, so extensive surgery with en bloc removal of the kidney was carried out. Intracanal extension of tumor thrombosis was not considered to be a contraindication to surgery. Studies have shown that maximal debulking has improved survival [10].

The decision to administer adjuvant chemotherapy is predominantly based on the risk of disease recurrence. Mitotane is the preferred agent in use [11]. Our patient had high-grade disease; hence, she was treated with adjuvant mitotane and was planned for continued surveillance for the next five years. The use of adjuvant radiotherapy has been less well-defined. Molecular targeted therapies against programmed death-ligand 1 (PD-LI), insulin-like growth factor 1 receptor (IGF-1R), vascular endothelial growth factor (VEGF), and epidermal growth factor (EGF) are being evaluated in phase I/II trials. Postoperative hormone assessment is essential to look for hypercortisolism or adrenal insufficiency. In cases of hormonal excess, the addition of steroidogenic enzyme inhibitors, such as metyrapone or ketoconazole, is recommended.

The prognosis depends on the clinical stage at presentation and the completeness of resection. Five-year actuarial survival was 66%, 58%, 24%, and 0% for stage I, II, III, and IV diseases, respectively [9]. Other factors of prognostic value include histology (revised Weiss criteria), mitotic rate, and K1-67 expression. Our patient had a revised Weiss score of 4 and a mitotic rate of 46 per 50 HPF. Despite maximal debulking surgery, our patient had a five-year survival rate of 24%.

## Conclusions

Although adrenal adenomas comprise most of the adrenal incidentalomas noted on imaging, ACC needs to be in the differential diagnosis despite its rarity of presentation. Clinical presentation can occur in any decade of life, with a rapid onset of Cushing’s syndrome and virilization pointing a clue toward its diagnosis. To our knowledge, this is the first case of an adrenal incidentaloma presenting with a coexisting pulmonary embolism along with rapid-onset Cushing’s syndrome. ACC presenting as a peripheral pulmonary embolism whether due to tumor embolization or secondary to an underlying hypercoagulable state remains rare. Surgical resection remains the only long-term curative option.
